# Molecular insights into the modulation of the 5HT_2A_ receptor by serotonin, psilocin, and the G protein subunit Gqα

**DOI:** 10.1002/1873-3468.15099

**Published:** 2025-01-26

**Authors:** Niklas Viohl, Ali Asghar Hakami Zanjani, Himanshu Khandelia

**Affiliations:** ^1^ PHYLIFE, Physical Life Science, Department of Physics, Chemistry and Pharmacy University of Southern Denmark Odense Denmark; ^2^ Present address: Max Perutz Labs Vienna BioCenter, University of Vienna and Medical University of Vienna Vienna Austria

**Keywords:** 5HT_2A_R, free energy calculations, G protein‐coupled receptors, molecular dynamics simulations, psilocin, psychedelics

## Abstract

5HT_2A_R is a G‐protein‐coupled receptor that drives many neuronal functions and is a target for psychedelic drugs. Understanding ligand interactions and conformational transitions is essential for developing effective pharmaceuticals, but mechanistic details of 5HT_2A_R activation remain poorly understood. We utilized all‐atom molecular dynamics simulations and free‐energy calculations to investigate 5HT_2A_R's conformational dynamics upon binding to serotonin and psilocin. We show that the active state of 5HT_2A_R collapses to a closed state in the absence of Gqα, underscoring the importance of G‐protein coupling. We discover an intermediate “partially‐open” receptor conformation. Both ligands have higher binding affinities for the orthosteric than the extended binding pocket. These findings enhance our understanding of 5HT_2A_R's activation and may aid in developing novel therapeutics.

**Impact statement**

This study sheds light on 5HT_2A_R activation, revealing intermediate conformations and ligand dynamics. These insights could enhance drug development for neurological and psychiatric disorders, benefiting researchers and clinicians in pharmacology and neuroscience.

## Graphical abstract

We explore 5HT_2A_ receptor activation, a key target for psychedelic drugs, using molecular dynamics simulations. Without Gqα, the active receptor collapses to an inactive conformation, underscoring the G‐protein's role. Discovery of an intermediate “partially‐open” state and the higher affinity of ligands to the orthosteric binding site provides valuable insights into receptor function, useful for future therapeutics for mental health treatments.

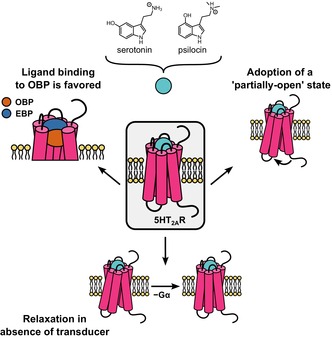

## Abbreviations

**5HTR**, serotonin receptor

**EBP**, extended binding pocket

**ECL**, extracellular loop

**GPCR**, G protein‐coupled receptor

**ICL**, intracellular loop

**MD**, molecular dynamics

**OBP**, orthosteric binding pocket

**PCA**, principal component analysis

**PMF**, potential of mean force

**RMSD**, root‐mean‐square distance

**RMSF**, root‐mean‐square fluctuation

**SEP**, side‐extended pocket

**TM**, transmembrane helix

**β**_**2**_ **AR**, β_2_‐adrenergic receptor

Neuronal signal transduction is achieved through an intricate interplay between various neurotransmitters and their corresponding postsynaptic receptors. Neurotransmitter systems in the brain include noradrenergic, dopaminergic, histaminergic, cholinergic, adrenergic, and serotonergic pathways. Among other things, serotonin signaling regulates mood, memory, sexual behavior, sleep, learning, and neural processing of cues for immediate behavioral responses and disruption of serotonergic pathways can promote numerous physical and mental disorders such as depression, migraine, schizophrenia, and anxiety [1, 2].

One promising group of rediscovered novel antidepressant candidates is psychedelic drugs such as psilocybin, MDMA, or LSD. Natural psychedelic compounds such as psilocybin or mescaline have been used by various indigenous cultures for centuries, usually integrating them into the framework of ritualistic contexts. Psilocybin occurs in a large variety of fungi known as “magic mushrooms.” In the liver, the prodrug psilocybin is rapidly converted into the pharmacologically active psilocin. After the chemical extraction and synthesis of psilocin, LSD, and other psychedelics in the 1950s and 1960s, pharmacological and behavioral research flourished until substance regulations diminished research activity [3, 4, 5]. Several recent clinical studies showed that the administration of hallucinogenic drugs can drastically improve mental disorders, while scarcely causing side effects and adverse reactions [6, 7, 8].

Following the release into the synaptic cleft, serotonin, and other effectors bind to one of the G protein‐coupled receptor (GPCR)‐type serotonin receptors (5HTRs) and stimulate a transducer‐mediated signaling pathway. The 5HT_2A_R receptor is believed to be responsible for psychedelic effects, though recent evidence suggests it is not directly or only partially involved in antidepressant effects [5, 9, 10, 11]. The binding of serotonin and other stimulants to 5HT_2A_R induces conformational changes in the intracellular region, which allow for the binding of the heterotrimeric Gq protein complex. Separately, β‐arrestin can be recruited through interactions with the phosphorylated disordered C‐terminal tail of 5HT_2A_R and binds to a distinct binding cavity [12, 13]. Ultimately, both transducer pathways alter gene expression patterns and kinase signaling, which in turn stimulate several neuroplastic processes [14, 15].

Due to the comparatively nonspecific binding mode, many psychedelics such as LSD and psilocin act promiscuously on many neural receptors [5]. Besides 5HT_2A_R, psilocin, and synthetic psilocin analogs exhibit binding affinities for 5HT_1A_R and 5HT_2C_R [16, 17, 18] and animal studies suggest that psilocin unfolds its psychedelic and antidepressant effects not solely via stimulation of 5HT_2A_R [19, 20, 21]. The structure of 5HT_2C_R in complex with psilocin shows that psilocin is similarly anchored in the orthosteric binding pocket (OBP) via the conserved central D134^3.32^ residue, and the indole core is coordinated through interactions with additional residues in the OBP [22]. Moreover, psychedelics such as ketamine, LSD, and psilocin influence synaptic plasticity, thereby facilitating antidepressant effects by binding to the neurotrophic tyrosine kinase receptor 2 (TrkB) and stimulating endogenous brain‐derived neurotrophic factor (BDNF) signaling [10, 11]. It was recently demonstrated that a 5HT_1A_R‐selective analog of the psychedelic substance 5‐MeO‐DMT analog lacks hallucinogenic effects but maintains anxiolytic and antidepressant activities in socially defeated mice [23].

Recent structural studies of 5HT_2A_R and other serotonin receptors identified binding modes of various agonists, inverse‐agonists, and antagonists (reviewed in Duan *et al*. [15] and Simon *et al*. [24]) as well as conformational changes during activation and transducer‐binding [22, 25, 26, 27, 28]. The binding pocket of 5HT_2A_R consists of two adjacent subpockets: the OBP and the extended binding pocket (EBP) (Fig. 5). Regardless of subpocket localization, ligands are universally anchored in the binding pocket by the central D155^3.32^ residue through interactions with a charged amine moiety [25, 26, 29, 30, 31]. Additional ligand groups are typically coordinated through hydrophobic interactions and hydrogen bonds by residues in the OBP and EBP. Crystal structures of 5HT_2A_R in complex with serotonin and psilocin showed that the indole core is exclusively located in the EBP, while the OBP and side‐extended pocket (SEP) are occupied by a lipid molecule [30]. On the contrary, computational studies on 5HT_2A_R [25, 32] as well as structural studies on related 5HTRs [22, 27, 28] showed an occupation of the OBP by the indole core of serotonin or psilocin. Mutational studies revealed that disruption of coordination in both subpockets diminishes receptor activity, hinting at two binding poses [30]. Antagonists typically extend beyond the EBP/OBP and additionally reach into the deep binding pocket, where they prevent necessary receptor rearrangements [29, 30].

Activation of 5HT_2A_R is marked by an extensive outward tilt of TM5 and TM6 accompanied by a smaller inward tilt of TM2 and TM3. This TM repositioning in the intracellular part of 5HT_2A_R opens the transducer binding cavity and allows Gqα or β‐arrestin to bind. Besides the large helix movements, 5HT_2A_R activation is also marked by structural changes in several functional motifs along the receptor: the rotation of the toggle switch residue W336^6.48^, movement in the PIF motif (P246^5.50^, I163^3.40^, and F332^6.44^), the breaking of the ionic lock between R173^3.50^ and E318^6.30^ as well as the inward shift of the NPXXY motif (N376^7.49^, P377^7.50^, and Y380^7.53^) accompanied by the breaking of the π‐stacking interactions between Y380^7.53^ and Y387^8.50^ [25, 26].

In this contribution, we use molecular dynamics (MD) simulations and free energy calculations to examine the details of the binding of psilocin and serotonin to the two different binding pockets and find that both ligands bind to the OBP with higher affinity. We also investigate the conformational transitions of the receptor and find that the active state is only stable with bound Gqα. We also discover a new conformation where the transitions described in the previous paragraph remain incomplete. We call this the partially‐open state of the receptor. MD simulations have been extensively used to derive dynamic and molecular details of serotonin receptors [33, 34].

## Materials and methods

### System construction and equilibration

**Fig. 1:**
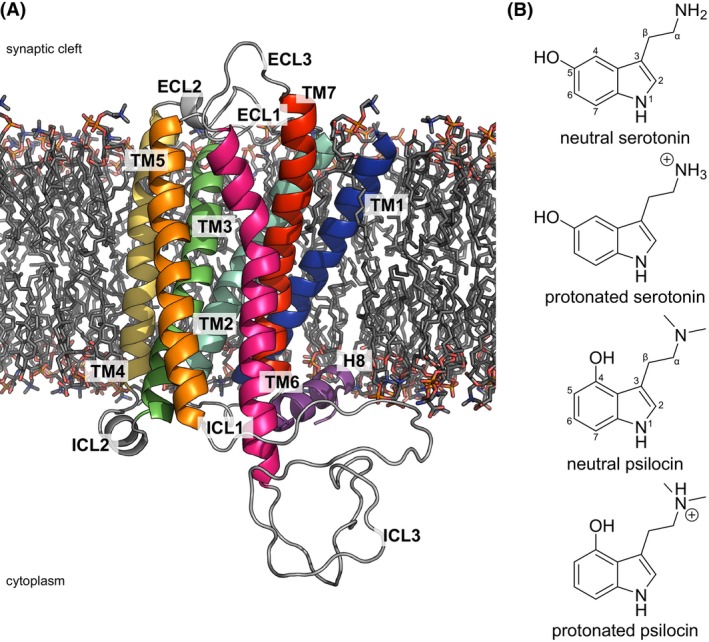
Structures of the simulation model system and ligands. (A) Structure of a 5HT_2A_R (70–399) model inserted into the simplified postsynaptic neural membrane. 5HT_2A_R has a typical GPCR structure with seven transmembrane helixes connected by intrinsically disordered extracellular and intracellular loops. Water molecules and ions are omitted for clarity. (B) Chemical structure of serotonin and psilocin. The basic α‐amine group of serotonin (p*K*_a_ 9.97) and psilocin (p*K*_a_ 8.47) is likely to be protonated under physiological conditions [53].

Experimental protein models of 5HT_2A_R (active state PDB: 7RAN/6WHA; inactive state PDB: 6A93) and the Gqα (AlphaFold database; PDB: 7W40) subunit were taken from the Protein Data Bank [35] as well as the AlphaFold Protein Structure Database [36, 37] and missing side chain atoms were filled in using pymol (Schrödinger, LLC: New York, NY, USA). A detailed description of utilized software and the different systems is given in Tables S1 and S2, respectively. Unresolved ICLs and ECLs were modeled as disordered loops using modeller 10.4 [38, 39] according to the canonical amino acid sequence of human 5HT_2A_R (UniProtKB accession number: P28223‐1). Final receptor models consist of residues 70–399 and contain TM1‐TM7, H8, ECL1‐ECL3, and ICL1‐ICL3 (Fig. 1A). Due to the lack of experimental structures, ligands were placed into the OBP according to previous computational studies of 5HT_2A_R [32]. In short, ligands were pulled from the aqueous phase toward the center of mass of the ligand binding pocket and subsequently repositioned in successive short restrained and restraint‐free simulations. Final ligand orientations were similar to the orientation of ligands in experimental 5HT_1_R structures [27]. For the EBP, ligands were placed according to the experimental crystal structures of 5HT_2A_R (PDBs: 7WC4/7WC5) [30]. A detailed overview of all systems is given in Table S2.

Assembled protein systems were oriented using the ppm 3.0 Web Server [40] and constructed using the Membrane Builder tool of charmm‐gui [41, 42]. Protein models were inserted into a heterogeneous lipid bilayer membrane consisting of 400 lipids in total with an extracellular leaflet composition of 30% POPC (16:0/18:1), 12% PSPC (16:0/18:0), 9% PAPC (16:0/20:4), 9% SDPC (18:0/22:6), 10% SSM, 30% CHL and an intracellular leaflet composition of 30% POPC (16:0/18:1), 12% PSPC (16:0/18:0), 9% PAPC (16:0/20:4), 9% SDPC (18:0/22:6), 20% POPS (16:0/18:1), 20% CHL, mirroring a simplified lipid composition of a postsynaptic neural membrane [43, 44, 45]. All systems were solvated with TIP3P water with Van der Waals interactions on hydrogen atoms, neutralized with K^+^/Cl^−^ counterions, and adjusted to an ionic strength of 150 mm KCl using standard gromacs tools. Initial box sizes and the number of water molecules for the different systems are listed in Table S2.

Systems were energy minimized using the steepest‐descent algorithm to remove steric clashes in the initial model. After minimization, systems were equilibrated for 250 ps in an NVT ensemble and 1625 ps in an NPT ensemble using velocity‐rescaling temperature coupling [46] and semi‐isotropic stochastic cell rescaling pressure coupling [47], while successively reducing position restraints. All simulations were carried out using GROMACS 2023.3 [48] and the CHARMM36 force field (version JUL2022) [49, 50]. A more detailed description of utilized parameters for production runs is given below. Topologies and force field parameters for protonated serotonin, protonated psilocin, and guanosine‐5′‐diphosphate (GDP) were generated using the CHARMM General Force Field (CGenFF) program and converted to GROMACS format using the CHARMM36 force field (version FEB2021) and the python script (cgenff_charmm2gmx_py2_nx1.py) from the MacKerell laboratory [51, 52]. Charge penalty determination and parameter validation were carried out in previous studies and were adopted for simulations in this study [53, 54]. The basic α‐amine group of serotonin and psilocin as well as D155^3.32^ in the ligand binding pocket can be protonated or deprotonated under physiological conditions. Previous simulations showed that the interaction between deprotonated anionic D155^3.32^ and protonated cationic serotonin and psilocin is the most stable configuration [32]. Thus, all simulations were carried out with deprotonated D155^3.32^ and protonated serotonin and psilocin, respectively (Fig. 1B).

### Conventional molecular dynamics simulations

For production, systems without Gqα were simulated for a total of 2 μs and systems with Gqα for a total of 1 μs with a time‐step of 2 fs using the leap‐frog algorithm. Three replicas of each were simulated with different initial velocity distributions during the first step of equilibration. Temperature and pressure were kept constant at 310 K and 1 bar using the Nosé–Hoover thermostat [55, 56] and the semi‐isotropic Parrinello‐Rahman barostat [57]. The Verlet neighbor search algorithm [58] was used to update the neighbor list, hydrogen bonds were constrained using the Linear Constraint Solver (LINCS) algorithm [59], van der Waals interactions were smoothly switched off between 1.0 and 1.2 nm, and electrostatic interactions were treated with the particle mesh Ewald (PME) method [60, 61].

Trajectories were visually inspected using Visual Molecular Dynamics (VMD) [62]. To monitor extensive conformational changes, the RMSD of the protein backbone and the RMSF of every residue were calculated with respect to the aligned initial structure using standard GROMACS tools. Frames every 0.1 ns were analyzed for RMSD and RMSF calculation. Conformational changes associated with 5HT_2A_R activation were assessed using intramolecular distances, the A100 index for class A GPCR activation [63], and principal component analysis (PCA). Distances between Cα atoms of R173/E318 and Q262/E318 were determined throughout the simulations using standard GROMACS tools. Reference distances for the “inactive” and “active” states were derived from the respective experimental structures. For the time evolution of the A100 score

(1) $$A100\left(5{\mathrm{HT}}_{2A}R\right)=\begin{array}{l} -14.43r\left(V95^{1.53}-L382^{7.55}\right)\\ -\;7.62r\left(D120^{2.50}-T160^{4.42}\right)\\ +\;9.11r\left(H165^{3.42}-A192^{4.42}\right)\\ -\;6.32r\left(Q262^{5.66}-C322^{6.34}\right)\\ -\;5.22r\left(A346^{6.58}-L362^{7.35}\right)+278.88, \end{array}$$

time courses of Cα distances were extracted from the respective trajectory using standard GROMACS tools and converted according to Eqn (1) into the A100 score using a custom Python script. In the three‐state model of the A100 score, conformations with an A100 score < 0 are classified as “inactive”, conformations with an A100 score < 55 are classified as “intermediate”, and conformations with an A100 score > 55 are classified as “active”. The two‐state model divides receptor conformations into two classes “active” and “inactive” at an A100 score of 25 [63]. For PCA, first, mass‐weighted covariance matrices of the 5HT_2A_R backbone were calculated and diagonalized. Afterward, 2D projections of trajectories onto the first and second eigenvectors were calculated using standard GROMACS tools. Due to their strong inherent flexibility, ICLs (residues 102–108, 179–189, and 263–314) and ECLs (residues 138–145, 217–231, and 347–354) were omitted for analysis. Centroids of clusters correspond to representative conformational states and were determined using a *k*‐means clustering algorithm. For comparison, representative conformational states were aligned with the experimental “inactive” (PDB: 6A93) [29] and “active” (PDB: 7RAN) [26] state structures of 5HT_2A_R.

### Potential of mean force calculations

Binding free energies of psilocin and serotonin to the OBP and EBP (Fig. 5) as well as the C‐terminal helix of Gqα to the intracellular binding cavity (Fig. S7) in different states were determined through PMF calculations using umbrella sampling simulations [64]. The C‐terminal helix of Gqα is the main Gqα entity that contacts the receptor. Therefore, the binding free energy between the helix and the receptor is an excellent proxy for the overall receptor‐Gqα binding free energy (Fig. S7). Simulations of the helix instead of the entire Gqα save significant computational resources. Furthermore, protein–protein binding affinity measurements between large proteins along a distance reaction coordinate can suffer from the influence of other degrees of freedom such as protein rotation which can confound the PMF [65]. Systems were prepared and equilibrated as described above. Since the TMs of the “active” state model rapidly repositioned during conventional MD simulations, the positions of backbone atoms were restrained with a force constant of 100 kJ·mol^−1^·nm^−2^. The hydrogen bond network of the C‐terminal Gqα helix dissipated partly during simulations of most umbrella windows, so distance restraints between Cα atoms were used to maintain the proper secondary structure of the helix. Initial configurations along the reaction coordinate ξ, defined as the *z*‐axis (membrane normal), were created through steered molecular dynamics (SMD) simulations by pulling the ligands from the respective binding site for 500 ps with a force constant of 1000 kJ·mol^−1^·nm^−2^ and a rate of 0.01 nm·ps^−1^. The resulting 40 windows per simulation had a uniform spacing of ~ 0.1 nm over a total of ~ 4 nm. Regions with a lack of sampling were complemented with additional asymmetric windows according to the umbrella histograms. Each window was equilibrated for 1 ns and conformations were subsequently sampled for 100 ns/50 ns (ligand binding/Gqα binding), for a total simulation time of ~ 4 μs/~ 2 μs per system. The initial center of mass (COM) distance between the ligand and the binding site in every window was restrained with a force constant of 1000 kJ·mol^−1^·nm^−2^. Other simulation parameters were like the parameters of conventional MD simulations described above. PMF profiles were extracted with the Weighted Histogram Analysis Method (WHAM) [66] and statistical errors were estimated through Bayesian bootstrap analysis [67] using the GROMACS WHAM module [68].

## Results

### “Open” state 5HT_2A_R rapidly relaxes in the absence of Gqα

Recent structural studies of 5HT_2A_R produced multiple conformations during different stages of receptor activation. The structures of 5HT_2A_R in an active G protein‐bound “open” state as well as in an inactive risperidone‐bound “closed” state identified prominent changes in several structural motifs during activation. To capture dynamic structural changes between these endpoints of activation and the influence of agonists on these conformational changes, we performed long all‐atom MD simulations using the CHARMM36 force field. We started simulations from both the inactive “closed” and active “open” states and tested the influence of ligands by inserting serotonin and psilocin into the respective subpocket location.

During production, all systems are comparatively stable over time but show a high deviation from the initial structure with root‐mean‐square distance (RMSD) values of 5–10 Å, independent of the presence of serotonin or psilocin bound the extracellular ligand binding pocket (Fig. S1A). Root‐mean‐square fluctuations (RMSF) analysis shows that this deviation mostly arises from changes in the highly flexible intrinsically disordered intracellular loops (ICLs) and extracellular loops ECLs (Fig. S1B) and does not necessarily indicate regulatory conformational changes in the receptor.

**Fig. 2:**
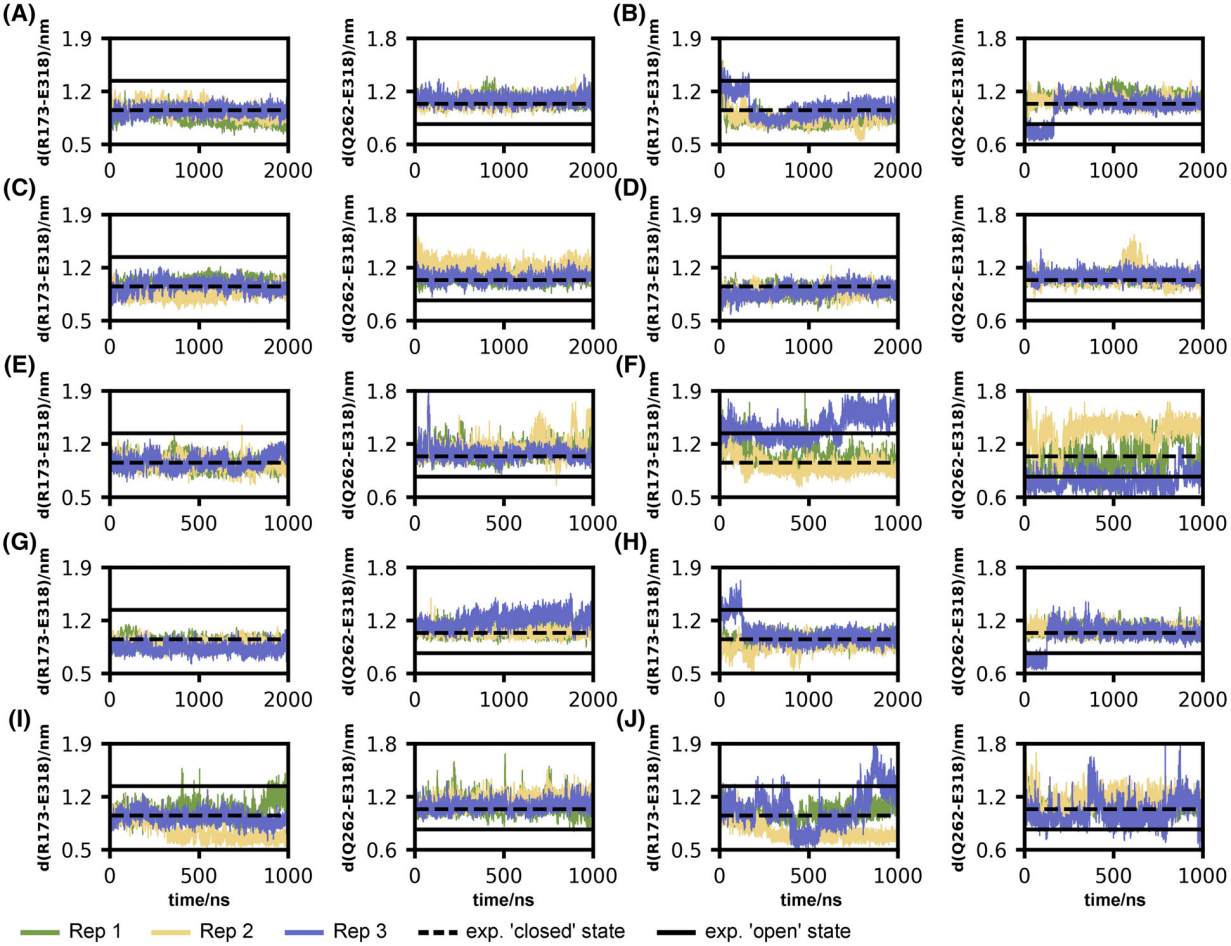
Changes in intramolecular Cα distances characterizing the opening of the intracellular transducer binding cavity through the outward shift of TM5 and TM6 during receptor activation throughout the simulation. (A) “Closed” 5HT_2A_R model without ligand. (B) “Open” 5HT_2A_R model without ligand. (C) “Closed” 5HT_2A_R model with psilocin in OBP. (D) “Open” 5HT_2A_R model with psilocin in OBP. (E) “Closed” 5HT_2A_R model with psilocin in EBP. (F) “Open” 5HT_2A_R model with psilocin in EBP. (G) “Closed” 5HT_2A_R model with serotonin in OBP. (H) “Open” 5HT_2A_R model with serotonin in OBP. (I) “Closed” 5HT_2A_R model with serotonin in EBP. (J) “Open” 5HT_2A_R model with serotonin in EBP. (A–J) The R173‐D318 distance also characterizes the breaking of the ionic lock. The horizontal dashed and solid lines are the experimental references for the inactive “closed” [29] and active “open” [25, 26] states. Distances in the presence of Gqα are depicted in Fig. S2.

To capture structural changes in the intracellular transducer binding cavity during transitions from the experimental “open” and “closed” states, we monitored distances between the Cα atoms of R173^3.50^/E318^6.30^ and Q262^5.66^/E318^6.30^ (Fig. 2)—assessing the outward tilt of TM5 and TM6. None of the inactive “closed” systems show an opening of the transducer binding cavity via outward movements of TM5 and TM6 as the distance remains at the level of the “closed” experimental reference [29]. On the other hand, the outward‐tilted TM5 and TM6 in the activated “open” state models rapidly collapse to an inward‐tilted TM5/TM6 orientation similar to the “closed” state during equilibration or within the first 250 ns of production. Only during one simulation replica did the receptor remain in the “open” state for the full simulation of 1 μs (Fig. 2F, blue). To test whether the helix conformation of the “open” state is stable in the presence of a transducer, we performed the same set of MD simulations and intramolecular distance analysis with a full Gqα subunit bound to the “open” intracellular transducer binding cavity and in direct proximity to the “closed” intracellular transducer binding cavity (Fig. S2). Except for one replica, the outward orientation of TM5 and TM6 in the “open” state is preserved and in good agreement with the experimental references, while the “closed” systems maintain the inward orientation of TM5 and TM6.

To further assess the activation state of 5HT_2A_R in an independent more generalized approach, we determined time evolutions of the A100 activation index for class A GPCRs [63] throughout our simulations of the receptor in the absence of a bound ligand and the presence of serotonin and psilocin (Fig. S3). Contrary to the rapid collapse of the “open” to a “closed” state assessed by the Cα distances of R173^3.50^/E318^6.30^ and Q262^5.66^/E318^6.30^ derived from the experimental structures of 5HT_2A_R, the A100 score indicates that the “open” conformation partly closes but remains in an intermediate state or fluctuates between an active and inactive state (Fig. S3, right) according to the three‐state model and two‐state model [63], respectively. Since the A100 only captures an overall activation state instead of single molecular aspects of GPCR activation, the intermediate conformation most likely adopts a conformational state in which TM5 and TM6 adopt an inward shifted state in the intracellular binding cavity while the rest of the receptor adopts an activated conformation, overall corresponding to the partially active intermediate state R″ [69]. Regarding the conformational states of the “closed” systems, both analytical approaches show that the “closed” conformation of 5HT_2A_R predominantly remains in an inactive closed conformation throughout our simulation on a large temporal scale (Fig. 2 and Fig. S3, left). The discrepancy between the two analysis approaches regarding the state of the simulations of the receptor in the “open” conformation underscores the importance of employing multiple methods to evaluate a complete picture of GPCR activation.

The rapid collapse of the “open” state of 5HT_2A_R during the simulations could imply that the large outward movements necessary for adopting the active state seen in structural studies [25, 26] depend on the presence of a bound transducer. Therefore, the dichotomy of an active and inactive state represents an incomplete conformational landscape of 5HT_2A_R activation. Before the final binding of a transducer, 5HT_2A_R probably either adopts an intermediate state in a local energy minimum or undergoes a gradual opening of the intracellular binding cavity without adopting a distinct intermediate conformation as seen for other GPCRs [70].

### 5HT_2A_R adopts “partially‐open” states during activation

**Fig. 3:**
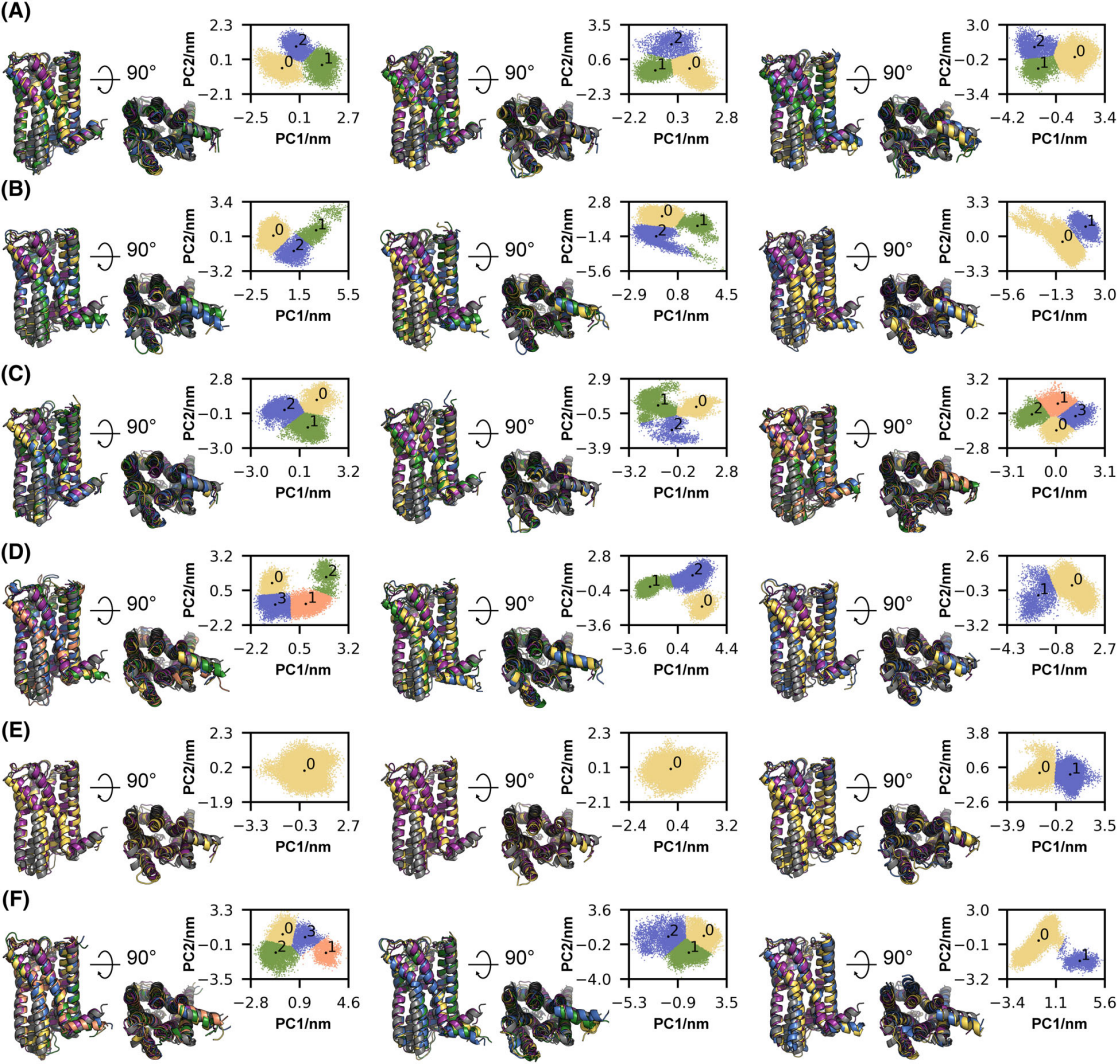
Principal component analysis of the conformational changes in the 5HT_2A_R backbone during simulation. (A) “Inactive” 5HT_2A_R model without ligand. (B) “Active” 5HT_2A_R model without ligand. (C) “Inactive” 5HT_2A_R model with PSIL in the OBP. (D) “Active” 5HT_2A_R model with PSIL in the OBP. (E) “Inactive” 5HT_2A_R model with SERO in the OBP. (F) “Active” 5HT_2A_R model with SERO in the OBP. (A–F) 2D projection of the three independent trajectories on each system's first and second eigenvectors. Due to the high conformational flexibility, ICLs and ECLs were omitted from the analysis. Centroid structures were determined using k‐means clustering and aligned to the experimental “inactive” (purple, PDB: 6A93) and “active” (gray, PDB: 7RAN) structures.

**Fig. 4:**
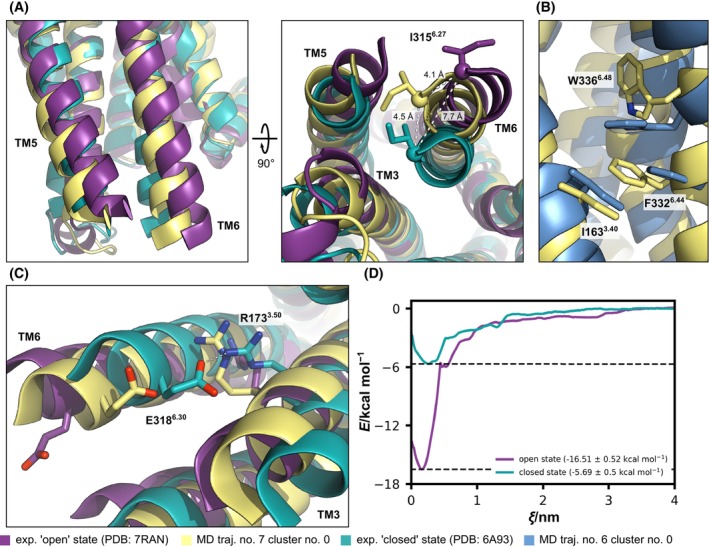
Comparison between the experimental “open”/Gq‐bound, the “partially‐open”, and the experimental “closed” states of 5HT_2A_R. (A) Outward movement of TM6. (B) Rotation of the toggle‐switch residue W336^6.48^ and the rearrangement of F332^6.44^ in the PIF motif. (C) Partial breaking of the ionic lock between R173^3.50^ and E318^6.30^. (A–C) An overview of all PCA clusters is depicted in Fig. 3. (D) PMF profiles for determining the binding free energy of the C‐terminal helix of Gqα to the intracellular transducer binding cavity. Profiles show the detachment of the C‐terminal helix of Gqα into bulk water for both the experimental active “open” and inactive “closed” states. For simplicity, the free energy of the system is considered zero when ligands are in bulk water. Data are represented as mean ± SD. Histogram overlaps are depicted in Fig. S4. A depiction of the interaction between 5HT_2A_R and the C‐terminal α5‐helix of Gqα in depicted in Fig. S7.

To determine more subtle conformational changes and protein motions in the transmembrane domain of 5HT_2A_R, we performed PCA on the backbone atoms of the TM1‐7/H8 core. Since the intrinsically disordered connecting loops are much more flexible than the TM1‐7/H8 core, and would otherwise dominate the eigenvalue spectrum, ICLs and ECLs were omitted for PCA. For each replica of the systems, up to four conformational ensembles were assigned using *k*‐means clustering, and representative conformations were aligned with the experimental “closed” and “open” state structures of 5HT_2A_R (Fig. 3) [25, 26, 29]. Unsurprisingly, the most prominent protein movement occurs in the intracellular short helix H8, which is connected to TM7 only via a short flexible linker and stabilized through π‐stacking interactions between Y380^7.53^ and Y387^8.50^. Despite the conformational diversity in this region, none of the ensembles exhibit an inward shift in the NPXXY motif comparable to the Gq‐bound experimental structures [25, 26] and are mostly conserved or even strengthened. As seen in the intramolecular distance analysis, the PCA shows that the active “open” systems predominantly adopt a “closed” transducer binding cavity conformation in the absence of Gqα, and the inactive “closed” systems retain their “closed” transducer binding cavity. However, in ~ 55% of the simulations, a small subsection of the conformational ensembles adopts a “partially‐open” state with a less extensive outward tilt of TM6 of ~ 4 Å instead of the ~ 8 Å in the experimental transducer‐bound “open” state (Fig. 4A) [25, 26]. These “partially‐open” states could represent an active conformation before Gqα binding that undergoes further opening of the intracellular binding cavity induced by proximity of a transducer molecule. Although the partial outward movement is reminiscent of the activated “open” state of the receptor, the “partially‐open” conformations lack other molecular characteristics of activation. Only two of the partially open conformational states undergo a rotation of the toggle switch residue W336^6.48^ and there are no subsequent rearrangements of F332^6.44^ in the PIF motif (Fig. 4B). Moreover, the ionic lock between R178^3.55^ and E318^6.30^ is loosened and not fully broken in most conformations. While the salt bridge between the two residues is broken, the side chains remain in comparatively close proximity so that a reformation of the ionic lock is likely (Fig. 4C). Notably, the partial opening of the transducer binding cavity occurs in the absence of ligands as well as in the presence of both serotonin and psilocin.

The general activation of GPCRs and the subsequent binding of the G protein transducer is marked by a sequence of conformational changes resulting in distinct intermediate states. The transition from the inactive ground state R to the inactive low‐affinity agonist‐bound state R′ is accompanied by only minor agonist‐induced conformational changes in the extracellular ligand binding pocket. Subsequently, the transition from the inactive low‐affinity agonist‐bound state R′ to the partially active intermediate state R″ is marked by a partial exposure of the intracellular transducer binding cavity through global rearrangements of TMs and rearrangements in distinct microswitch residues. Finally, the activated transducer‐bound states R* and R*^G^ are marked through extensive global rearrangements in the TMs that fully open the intracellular transducer binding cavity. These rearrangements allow for the initial insertion of the C‐terminal α5‐helix of the Gα subunit in the activated state R* and the full engagement of the Gαβγ—complex fully assembled active state R*^G^. Notably, the final transition from the activated state R* to the fully assembled active state R*^G^ is accompanied by the release of GDP and therefore irreversible [69]. Our initial conformations in the inactive “closed” and active “fully open” states correspond to the inactive states R/R′ and the fully assembled active state R*^G^, respectively. Comparing the different activation states to our PCA cluster centroids (Fig. 3) to representative conformations of the states R, R′, R″, R*, and R*^G^ [69] shows that most clusters, those with high conformational similarity to the experimental “closed” state of 5HT_2A_R, match the ligand‐free and ligand‐bound inactive states R and R′. The conformation of our “partially‐open” states (Fig. 4A–C) aligns well with the intermediate not fully stabilized state R″ in accordance with the observed partially fulfilled molecular characteristics of activation for 5HT_2A_R described above. Other studies on G protein activation found similar “partially‐open” intermediate states for β_2_AR by combining time‐resolved cryo‐EM and NMR spectroscopy with MD simulations [70, 71].

To investigate the thermodynamic implications of the different conformational states on Gqα binding, we employed potential of mean force calculations to determine the binding affinity of the C‐terminal helix of Gqα to the intracellular binding cavity of 5HT_2A_R in the inactive “closed” and the active “open” states (Fig. 4D and Fig. S7). In previous research, the C‐terminal α5‐helix is often highlighted as a key region in studying G‐protein binding in computational models [72]. Similarly, in a detailed molecular dynamics investigation involving seven different GPCRs and three different G‐proteins, the N terminus of the G‐protein was omitted from the calculations because it protruded out of the GPCR cavity, is highly flexible, and usually engaged in intramolecular interactions with the Ras domain of the G‐proteins [73]. The binding affinity of the C‐terminal Gqα helix is substantially higher to the “open” conformation than to the “closed” conformation. Despite the rapid collapse of the “open” state in the absence of a bound transducer, this difference in binding affinity demonstrates the necessity of a “fully‐open” transducer binding cavity for effective Gqα binding. However, the C‐terminal Gqα helix still shows a sizeable affinity to the “closed” binding cavity of 5HT_2A_R with a binding free energy of ~ −10.8 kcal·mol^−1^. The initial low‐affinity binding may serve to align and tether both the receptor and transducer, facilitating the progression toward the subsequent complete opening of the transducer binding cavity, driven by the increasing binding free energy.

### The binding of serotonin and psilocin to the OBP is energetically favored over binding to the EBP

**Fig. 5:**
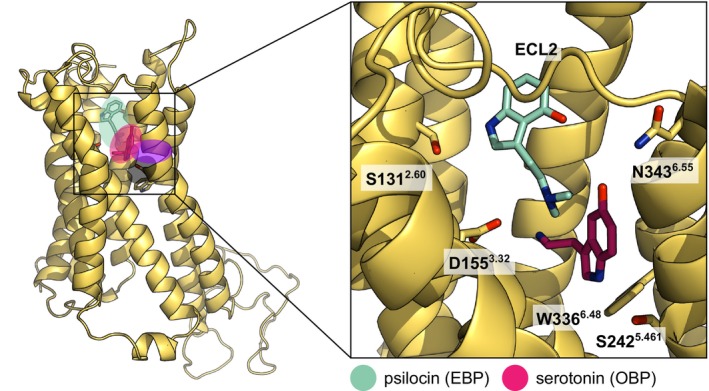
Overview of the extracellular ligand binding pockets of 5HT_2A_R. The receptor features four distinct binding pockets: the orthosteric binding pocket (OBP; magenta), the extended binding pocket (EBP; cyan), the deep binding pocket (DBP; gray), and the 5HT_2A_R‐specific side extended pocket (SEP; purple). Ligands are typically anchored in either binding pocket through interactions with the carboxyl group of residue D155^3.32^. The binding pocket is closed through a lid formed by the flexible ECL2 region. The magnification shows the position of psilocin in the EBP and serotonin in the OBP with highlighted key residues of 5HT_2A_R for efficient ligand binding.

The extracellular ligand binding pocket of 5HTRs consists of the upper EBP as well as the adjacent deeper OBP and is closed through a lid formed by ECL2 (Fig. 5). Due to the conserved G238^5.42^ residue in the 5HT_2_R family, the OBP is further extended in these receptors via a direct connection to the SEP. This extensive binding pocket allows small ligands such as serotonin and psilocin to adopt multiple binding modes by occupying the OBP and EBP, respectively [15, 24]. While the molecular basis of serotonin and psilocin binding has been investigated through structural, computational, and mutational studies [25, 30, 32], the underlying thermodynamic properties and dynamic processes between the two subpockets and their physiological implications remain unknown. Previous computational studies on GPCRs, especially on β‐adrenergic receptors, revealed multiple possible entry and exit pathways. In the dominant extracellular vestibule pathway, ligands enter the receptor between ECL2 and ECL3 and pass through a narrow cleft formed by TMs 5, 6, and 7 marked by distinct metastable intermediate conformations to reach the binding pocket [74]. Less frequently observed lateral pathways involve ligand movements through clefts formed by pairs of TMs [75].

**Fig. 6:**
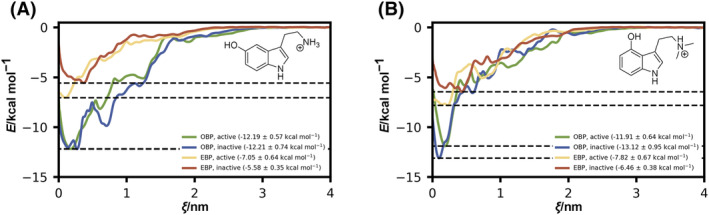
PMF profiles for the determination of the binding free energy of the agonists serotonin and psilocin to the extracellular ligand binding pocket. (A) Profiles show the detachment of serotonin from the OBP and EBP into bulk water for both the experimental active “open” and inactive “closed” states. Data are represented as mean ± SD. Histogram overlaps are depicted in Fig. S5. (B) Profiles show the detachment of psilocin from the OBP and EBP into bulk water for both the experimental active “open” and inactive “closed” states. Two hundred and fifty bootstraps for each system were used to calculate the free energy differences between the states when the compound was in the water phase and when it was in the binding site. The reported errors are standard deviations of these values. Histogram overlaps are depicted in Fig. S6. (A, B) For simplicity, the free energy of the system is considered zero, when ligands are in bulk water.

Thus, we performed potential of mean force calculations to determine the binding affinity of serotonin and psilocin to the two subpockets via the predominant entry/exit pathway through the extracellular vestibule and compared the affinities between the experimental “closed” and “open” transducer‐bound states (Fig. 6). In our simulations, the reaction coordinate was defined as the distance between the ligand and the binding pocket along the membrane normal (*z*‐axis), where the starting point represents the ligand bound to the respective binding pocket and the endpoint represents an unbound ligand in extracellular bulk water. We used 250 bootstraps for each system and calculated the free energy differences between the states when the compound was in the water phase and when it was in the binding site. The reported values are means and errors are standard deviations of these values. For both psilocin and serotonin, the binding free energy differs by ~ 5 kcal·mol^−1^ between the OBP and EBP (Table 1), hinting at a more stable binding mode in the deeper OBP as well as a higher relative occupancy of the OBP by psilocin and serotonin. Considering the probability distribution between the two binding modes using a simple Boltzmann factor

(2) $$\begin{array}{c} \frac{p_{\mathrm{OBP}}}{p_{\mathrm{EBP}}}=e^{\frac{\Delta G_{\mathrm{EBP}}-\Delta G_{\mathrm{OBP}}}{\mathrm{RT}}} \end{array}$$

derived from the differences in the binding free energies, the probability of an occupied OBP is higher by three orders of magnitude for the “open” state and four orders of magnitude for the “closed” state, respectively. During our conventional MD simulations, the ligands remained stably coordinated in the OBP throughout the full simulations, with ligands escaping the binding pocket in only two of 12 simulations. When placed in the EBP, ligands exhibit more dynamic coordination with movements toward the lid and escape the binding pocket in five of 12 simulations, further underlining the weaker affinity to this subpocket orientation. In the crystal structures of 5HT_2A_R, the occupation of the EBP is accompanied by the occupation of the OBP and SEP through a small monoolein lipid [30]. This occupation of the OBP/SEP through small lipids gives a molecular explanation for the observation that lipids act as partial agonists [30] and modulate 5HT_2A_R signaling [76, 77]. Our findings suggest that lipids like monoolein, oleamide, or 2‐oleoyl glycerol not only modulate 5HT_2A_R activity through competitive binding to the OBP but might be necessary to overcome the energy difference between the two binding modes for effective EBP‐mediated signaling.

**Table 1:** Binding free energies of psilocin and serotonin to the OBP and the EBP.

System	Δ*G*_OBP_ (kcal·mol^−1^)	Δ*G*_EBP_ (kcal·mol^−1^)	ΔΔ*G*_EBP‐OBP_ (kcal·mol^−1^)
SERO/open	−12.19 ± 0.74	−7.05 ± 0.64	5.14 ± 1.21
SERO/closed	−12.21 ± 0.74	−5.58 ± 0.35	6.63 ± 1.09
PSIL/open	−11.91 ± 0.64	−7.82 ± 0.67	4.09 ± 1.31
PSIL/closed	−13.12 ± 0.95	−6.46 ± 0.38	6.66 ± 1.33

Interestingly, there is no substantial difference in binding free energy between the “closed” and “open” states for the OBP. On the other hand, the lower binding free energy and the shift in the reaction coordinate of the energy minimum for the EBP in the “closed” state indicate that both ligands show a higher affinity to the EBP for the receptor in the activated “open” state. The binding of ligands to the EBP during the early stages of receptor activation, when the receptor still adapts a “closed” state, could induce conformational changes toward a more “open” state to accommodate for the lower binding free energy of the “closed” state. Moreover, the lower binding free energy of the EBP magnifies the difference in binding free energy between the two subpockets and therefore shifts the occupation of the two subpockets further toward the OBP for the “closed” state of the receptor. With this magnified difference for the inactive “closed” state, the occupation of the OBP by a lipid molecule might be particularly crucial for EBP‐mediated signaling during the early steps of receptor activation.

## Discussion

Using molecular dynamics simulations and free energy calculations, we investigated the binding dynamics of serotonin and psilocin to the 5HT_2A_R and the subsequent conformational changes during receptor activation. Our findings reveal several critical insights into the interaction mechanisms and structural transitions of 5HT_2A_R: serotonin and psilocin exhibit a higher binding affinity for the OBP over the EBP, which demonstrates stable ligand coordination in the OBP. In the absence of the Gqα subunit, 5HT_2A_R partly adopts a “partially‐open” state, characterized by less extensive outward movements of transmembrane helices compared to the fully “open” state observed with Gqα binding. This suggests that the receptor transitions through intermediate states essential for full activation. The active “open” state of 5HT_2A_R demonstrates a significantly higher affinity for the Gqα subunit compared to the “closed” state, emphasizing the importance of a fully open transducer binding cavity for effective Gqα interaction. Interactions between the receptor and the G‐protein's α1‐helix or βγ subunit could potentially affect the specificity of the receptor‐G‐protein coupling and are critical to understanding noncognate G‐protein binding [78, 79]. While our current focus was on the α5‐helix, future work could incorporate more detailed G‐protein models to explore these interactions in greater depth. In this study, we did not explicitly calculate how ligands influence G‐protein specificity and efficacy. However, it is well‐known that ligands can modify G‐protein coupling by stabilizing specific receptor conformations, which may, in turn, impact the efficacy of G‐protein signaling [80, 81, 82, 83]. This is a critical area for future research. Future studies could employ molecular dynamics simulations, alongside more detailed G‐protein models, to explore how different ligands affect receptor activation and G‐protein coupling efficiency. These findings will provide a more detailed mechanistic understanding of the binding dynamics and activation process of 5HT_2A_R, highlighting the significance of receptor‐ligand and receptor‐transducer interactions. Mechanistic insights from such investigations can guide the design of more selective and effective therapeutics targeting 5HT_2A_R for the treatment of a range of neuropsychiatric disorders, potentially leading to improved therapeutic outcomes with fewer side effects.

More recently, computational investigations of GPCRs have focused on drug design and have used the free energy perturbation (FEP) method or its variants for calculating the difference in the binding affinity of the native ligands, agonists, and antagonists [84, 85].

However, umbrella sampling has been used to shed light on the impact of binding of ligands to the interaction of GPCRs with the G‐protein, which is something that FEP calculations cannot directly address. Coarse‐grained simulations and umbrella sampling were used to show that the native ligand PIP2 (phosphatidylinositol 4,5‐bisphosphate) significantly stabilized the adenosine A2R‐mini‐Gs complex compared to PS (phosphatidylserine), by about 50 ± 10 kJ·mol^−1^ [86]. Remarkably, we find a similar difference in the free energy of binding between the receptor and C‐terminal helix of Gqα between the active and inactive forms of 5‐HT_2A_R. The presence of the G‐protein increased the binding affinity of the native ligand to the μ‐opioid receptor [87], most likely by stabilizing the active state of the GCPR. It is worth noting, however, that causality can work the other way, and the agonist is more likely to stabilize the state to which the G‐protein binds. In NMR investigations of the β_2_AR receptor bound to two different G‐proteins, it was shown that the binding of the agonist stabilized specific segments in TM4, TM5, TM6, and TM7, before coupling to the G‐proteins [88]. Enhanced sampling and umbrella sampling of the neurotensin receptor 1 with agonists, partial agonists, and antagonists showed that the orthosteric binding pocket undergoes dynamic expansions and contractions, while the G protein‐binding site transitions between inactive, intermediate, and active‐like states in response to ligand binding [89]. Umbrella sampling has recently been used to investigate the free energy of binding of adrenaline to the β_2_AR [90], but the impact on the binding of the G‐protein was not investigated. In a recent study about biased opioid receptor activation, PMF calculations were implemented to characterize distinct ligand binding modes in the μOR/δOR heterodimer thought to be implied in receptor efficacy [91].

We have observed the presence of both the closed and open states of the receptor without the ligand, although the open states rapidly collapse to the closed state. Along these lines, pressure‐resolved DEER spectroscopy was used to reveal the existence of both the inactive and active conformations within the equilibrium ensemble of the unliganded β_2_AR. This finding highlights the GPCR's intrinsic ability to sample active states even in the absence of a bound ligand [92].

Molecular dynamics simulations have become indispensable in advancing our understanding of GPCR dynamics, offering insights that are often beyond the reach of experimental techniques. Seminal studies such as [72, 74] have shown how MD can uncover ligand‐receptor binding pathways and G‐protein interactions, and our work extends this by exploring the conformational changes in the 5HT_2A_R when bound to serotonin and psilocin. This is in line with structural data obtained from cryo‐EM studies on GPCR‐G protein complexes further validating our computational observations of receptor activation and Gqα coupling.

The integration of MD simulations with experimental techniques is crucial for understanding receptor function. Our results align with experimental findings on serotonin signaling through 5HT_2A_R, a process extensively characterized by structural biology studies [82]. Moreover, the work of Hilger *et al*. [14] demonstrates how MD simulations can bridge gaps in experimental data, allowing us to visualize dynamic processes like receptor conformational changes and G‐protein binding in unprecedented detail.

## Author contributions

NV was involved in investigation, data curation, formal analysis, visualization, writing—original draft. AAHZ was involved in conceptualization, methodology, supervision, data curation, formal analysis, writing—review and editing. HK was involved in conceptualization, funding acquisition, supervision, project administration, writing—review and editing.

### Peer review

The peer review history for this article is available at https://www.webofscience.com/api/gateway/wos/peer‐review/10.1002/1873‐3468.15099.

## Supporting information


**Fig. S1.** Conformational changes during simulation of 5HT_2A_R.
**Fig. S2.** Changes in intramolecular Cα distances characterizing changes during receptor activation throughout the full 5HT_2A_R simulation in the presence of the transducer subunit Gqα, related to Fig. 2.
**Fig. S3.** Changes in A100 score during receptor activation throughout the simulation.
**Fig. S4.** Umbrella histograms for the PMF calculation of Gqα binding to the intracellular transducer binding cavity of 5HT_2A_R, related to Fig. 4D.
**Fig. S5.** Umbrella histograms for the PMF calculation of serotonin binding to the extracellular binding pocket of 5HT_2A_R, related to Fig. 6A.
**Fig. S6.** Umbrella histograms for the PMF calculation of psilocin binding to the extracellular binding pocket of 5HT_2A_R, related to Fig. 6B.
**Fig. S7.** Interactions between 5HT_2A_R and the C‐terminal α5 helix of Gqα, related to Fig. 4.
**Table S1.** Software and algorithms.
**Table S2.** Overview of system properties for MD simulations.

## Data Availability

All models, data, and scripts were deposited to the Zenodo repository and can be accessed via https://doi.org/10.5281/zenodo.12723328. Any additional information required to reanalyze the data reported in this paper is available from the lead contact upon request.
