# Supplementary material for: Molecular insights into the modulation of the 5HT 2A receptor by serotonin, psilocin, and the G protein subunit Gqα
Source: FEBS Lett. 2025 Jan 26;599(6):876–91. doi: 10.1002/1873-3468.15099 (PMC11931985; doi:10.1002/1873-3468.15099)
Supplement: Supplementary file 1 — Fig. S1. Conformational changes during simulation of 5HT2AR. Fig. S2. Changes in intramolecular Cα distances characterizing changes during receptor activation throughout the full 5HT2AR simulation in the presence of the transducer subunit Gqα, related to Fig. 2. Fig. S3. Changes in A100 score during receptor activation throughout the simulation. Fig. S4. Umbrella histograms for the PMF calculation of Gqα binding to the intracellular transducer binding cavity of 5HT2AR, related to Fig. 4D. Fig. S5. Umbrella histograms for the PMF calculation of serotonin binding to the extracellular binding pocket of 5HT2AR, related to Fig. 6A. Fig. S6. Umbrella histograms for the PMF calculation of psilocin binding to the extracellular binding pocket of 5HT2AR, related to Fig. 6B. Fig. S7. Interactions between 5HT2AR and the C‐terminal α5 helix of Gqα, related to Fig. 4. Table S1. Software and algorithms. Table S2. Overview of system properties for MD simulations. [file FEB2-599-876-s001.pdf]

Supporting Information for the Article

Molecular insights into the modulation of the $5HT_{2A}$ receptor by serotonin, psilocin, and the G protein subunit Gqα

Niklas Viohl, Ali Asghar Hakami Zanjani, and Himanshu Khandelia

This PDF contains

- Figures S1 to S7
- Tables S1 and S2

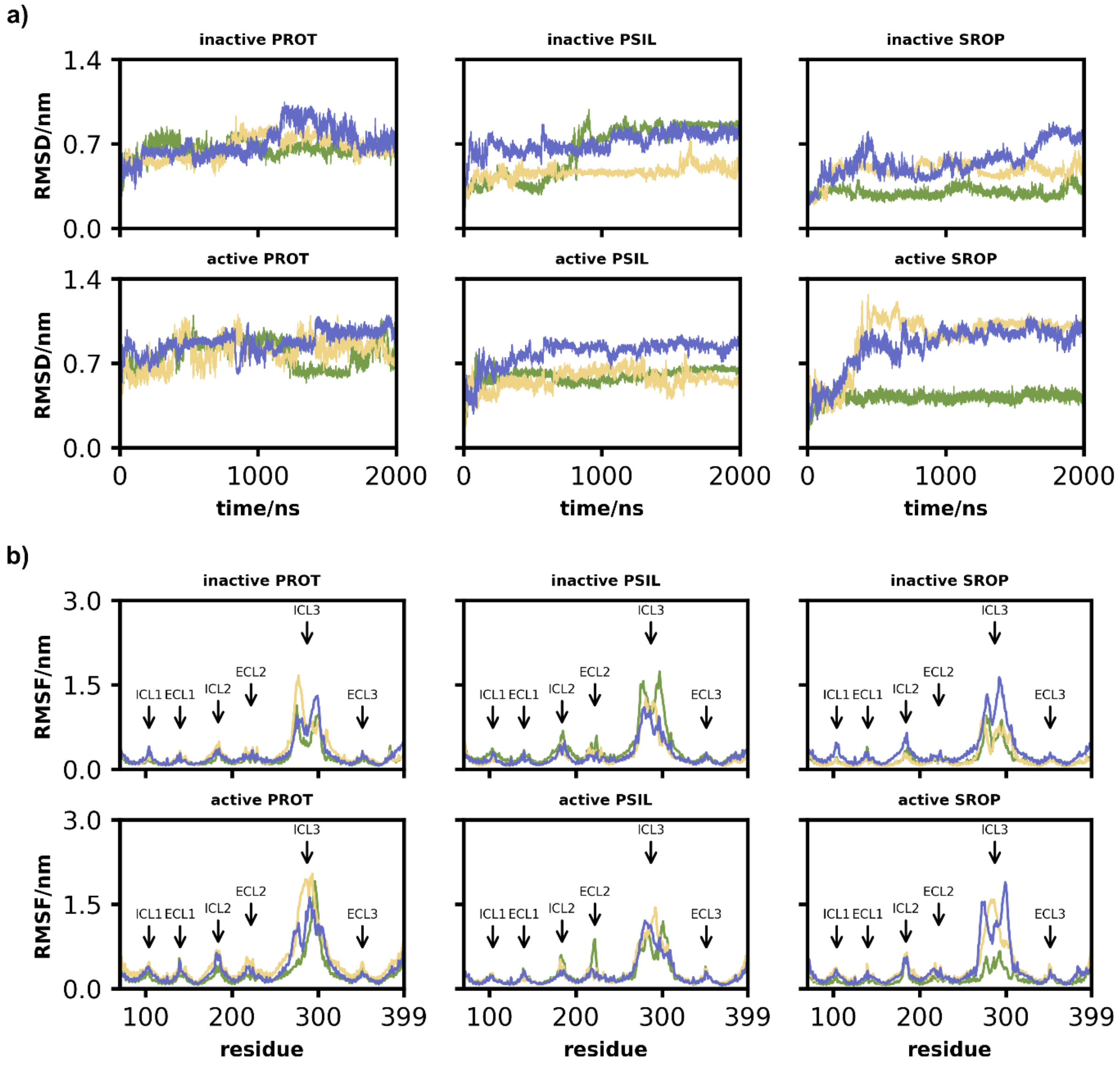

Figure S1. Conformational changes during simulation of 5HT$_{2A}$R. (A) RMSD of the 5HT$_{2A}$R backbone from the initial structure throughout the MD simulation. Time points every 0.1 ns were analyzed. (B) RMSF of 5HT$_{2A}$R residues from the initial structure throughout the MD simulation. The particular flexible loops between the TMs are marked.

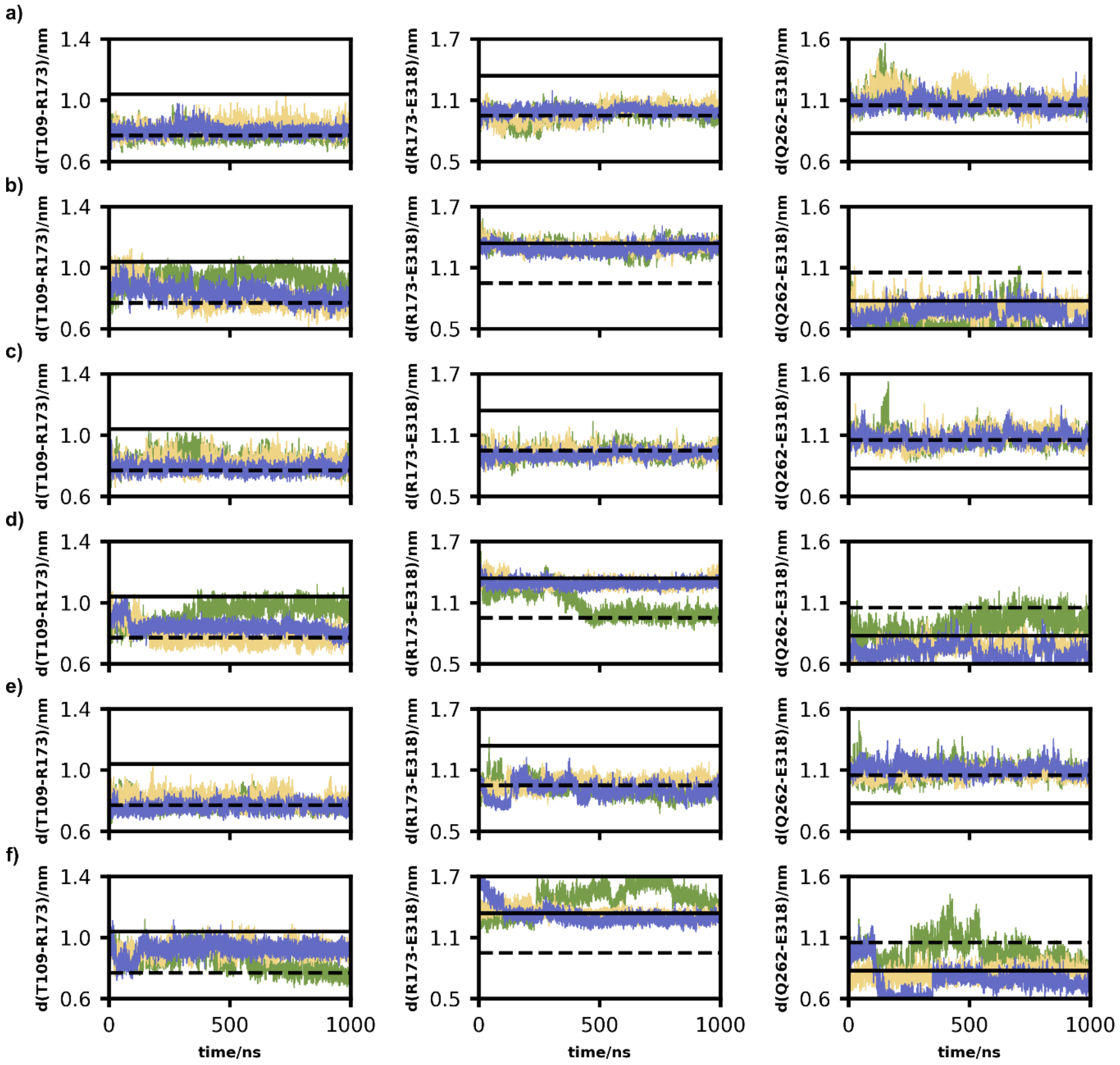

Figure S2. Changes in intramolecular Cα distances characterizing changes during receptor activation throughout the full $5HT_{2A}R$ simulation in the presence of the transducer subunit Gqα, related to Figure 2. (A) ‘Inactive’ $5HT_{2A}R$ model without ligand. (B) ‘Active’ $5HT_{2A}R$ model without ligand. (C) ‘Inactive’ $5HT_{2A}R$ model with PSIL in the OBP. (D) ‘Active’ $5HT_{2A}R$ model with PSIL in the OBP. (E) ‘Inactive’ $5HT_{2A}R$ model with SERO in the OBP. (F) ‘Active’ $5HT_{2A}R$ model with SERO in the OBP. (A–F) The outward shift of TM5 and TM6 is assessed through the R173-E318 distance and Q262-E318 distance, respectively. Additionally, the former also characterizes the breaking of the ionic lock. The inward shift of TM2 and TM3 is measured via the T109-R173. Dashed: Intramolecular distance in the experimental ‘inactive’ structure. Solid: Intramolecular distance in the experimental ‘active’ structure.

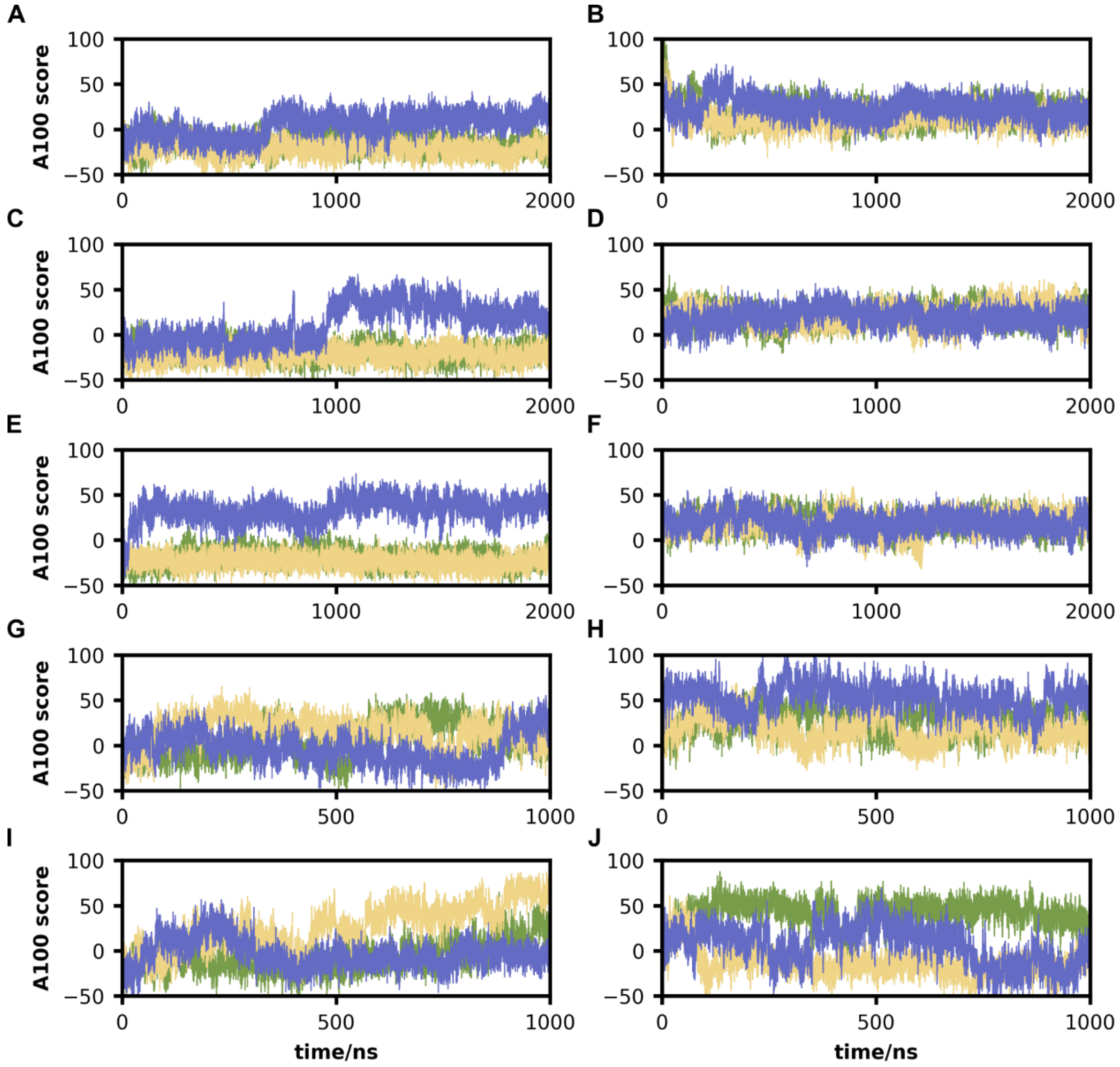

Figure S3. Changes in A100 score during receptor activation throughout the simulation. (A) ‘Inactive’ $5HT_{2A}R$ model without ligand. (B) ‘Active’ $5HT_{2A}R$ model without ligand. (C) ‘Inactive’ $5HT_{2A}R$ model with PSIL in the OBP. (D) ‘Active’ $5HT_{2A}R$ model with PSIL in the OBP. (E) ‘Inactive’ $5HT_{2A}R$ model with SERO in the OBP. (F) ‘Active’ $5HT_{2A}R$ model with SERO in the OBP. (G) ‘Inactive’ $5HT_{2A}R$ model with PSIL in the EBP. (H) ‘Active’ $5HT_{2A}R$ model with PSIL in the EBP. (I) ‘Inactive’ $5HT_{2A}R$ model with SERO in the EBP. (J) ‘Active’ $5HT_{2A}R$ model with SERO in the EBP. (A-J) Green: replica 1; yellow: replica 2; blue: replica 3.

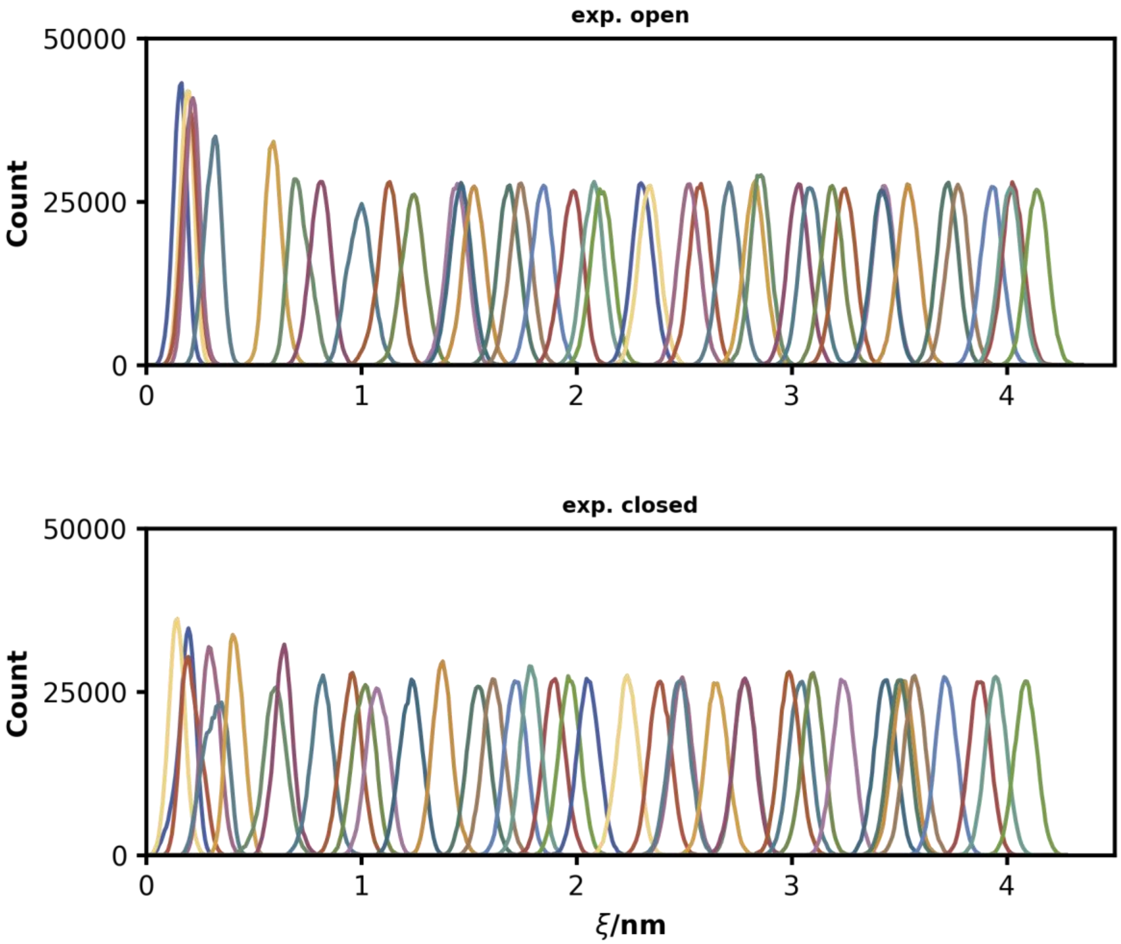

Figure S4. Umbrella histograms for the PMF calculation of Gqα binding to the intracellular transducer binding cavity of $5HT_{2A}R$, related to Figure 4D.

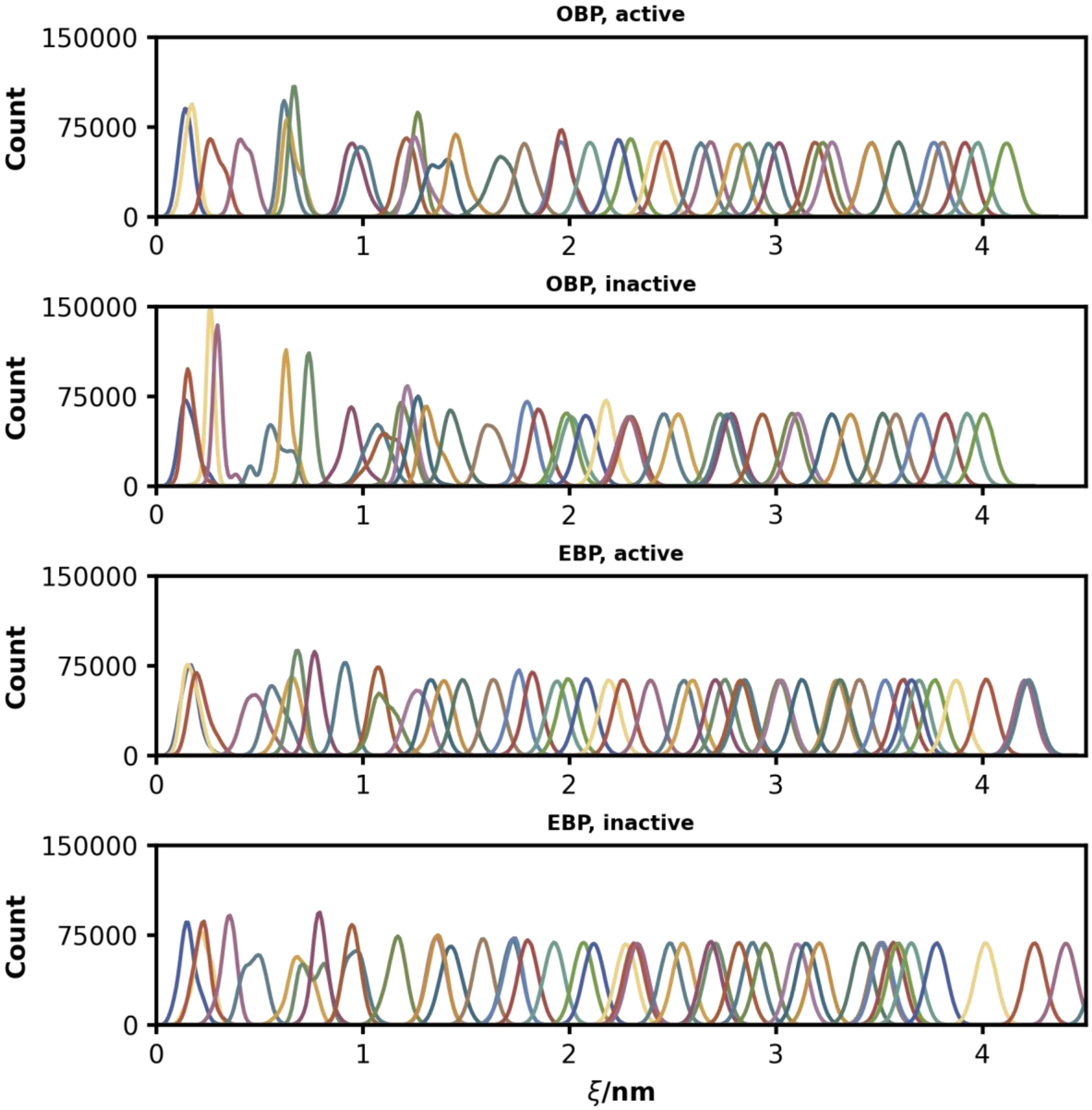

Figure S5. Umbrella histograms for the PMF calculation of serotonin binding to the extracellular binding pocket of $5HT_{2A}R$, related to Figure 6A.

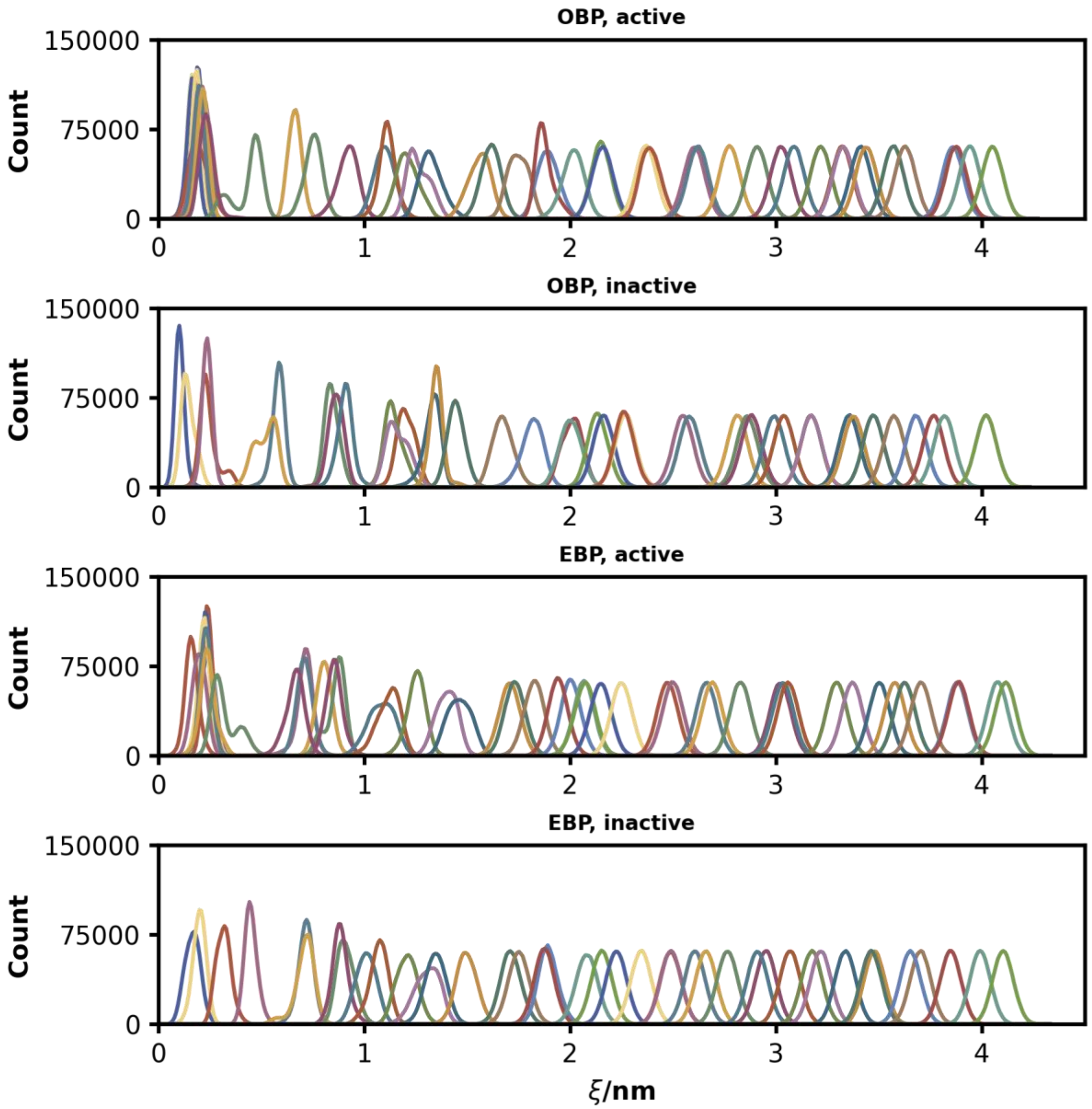

Figure S6. Umbrella histograms for the PMF calculation of psilocin binding to the extracellular binding pocket of $5HT_{2A}R$, related to Figure 6B.

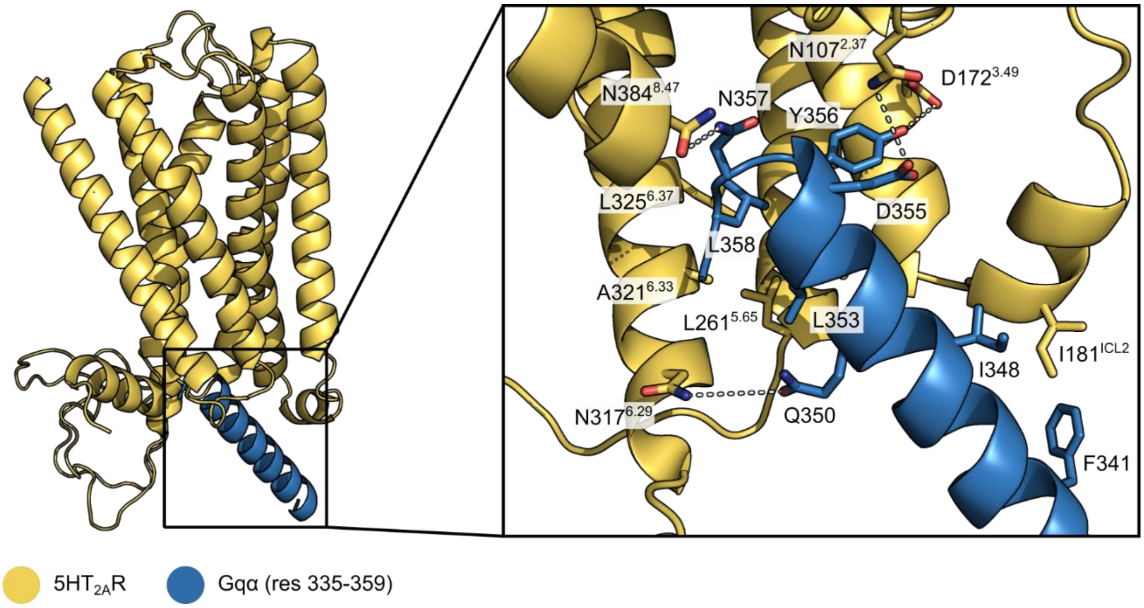

Figure S7. Interactions between 5HT$_{2A}$R and the C-terminal α5 helix of Gqα, related to Figure 4. The interaction between the intracellular transducer binding cavity and the α5 helix of Gqα is mediated through multiple polar interactions with residues in TM 2, 3, 5, 6, and 8. Furthermore, TMs 5 and 6 form a large hydrophobic patch interacting with leucine residues at the distal end of the Gqα helix.

Table S1. Software and algorithms

Software/Algorithm	Version	Purpose	Reference/Source
Python3	3.x	Used for custom scripting, data processing, and analysis of molecular dynamics simulation data.	https://www.python.org
Visual Studio Code	Latest	Integrated development environment (IDE) used for coding, debugging, and writing Python scripts.	https://code.visualstudio.com
PyMOL	2.6	Molecular visualization software used to analyze and generate structural figures for proteins.	https://pymol.org
Visual Molecular Dynamics (VMD)	Latest	Used for visualizing molecular dynamics trajectories and analyzing simulation results.	https://www.ks.uiuc.edu/Research/vmd
MODELLER	10.4	Employed for homology or comparative modeling of protein 3D structures based on known templates.	https://salilab.org/modeller
PPM 3.0 Web Server	3.0	Prediction of membrane protein structures and positioning of proteins within the lipid bilayer.	https://opm.phar.umich.edu/ppm_server
CHARMM-GUI	Latest	Used to prepare molecular systems, including membrane bilayers, for MD simulations with GROMACS.	https://www.charmm-gui.org
GROMACS	2023.3	Molecular dynamics simulation engine used for running and analyzing biomolecular simulations.	https://www.gromacs.org
CHARMM36 Force Field	JUL2022	The force field applied for simulating proteins and lipids in MD simulations, providing parameters for accurate molecular modeling.	https://mackerell.umaryland.edu/charmm_ff.shtml#gromacs
CGenFF	4.6	Applied to simulate small molecules in conjunction with CHARMM36 for accurate free energy calculations.	https://cgenff.com
Protein Data Bank	-	Database repository of experimentally determined 3D structures of proteins and nucleic acids.	https://www.rcsb.org
AlphaFold Database	-	Used to retrieve predicted 3D protein structures based on sequence data.	https://alphafold.ebi.ac.uk/
UniProt	-	Protein sequence database used for obtaining detailed annotations on protein sequences.	https://www.uniprot.org/

Table S2. Overview of system properties for MD simulations.

$5HT_{2A}R$ model	ligand	transducer	box size/Å^3	no. water	no. lipids	traj. no.
Conventional MD simulations						
7RAN*	—	—	115×115×147	~43000	400	4, 5, 6
7RAN*	$SERO^+$ (OBP)	—	105×105×161	~43000	400	10, 11, 12
7RAN*	$PSIL^+$ (OBP)	—	105×105×161	~43000	400	16, 17, 18
6WHA*	$SERO^+$ (EBP)	—	115×115×157	~53000	400	22, 23, 24
6WHA*	$PSIL^+$ (EBP)	—	115×115×157	~53000	400	28, 29, 30
6A93†	—	—	115×115×163	~50000	400	1, 2, 3
6A93†	$SERO^+$ (OBP)	—	105×105×183	~50000	400	7, 8, 9
6A93†	$PSIL^+$ (OBP)	—	105×105×183	~50000	400	13, 14, 15
6A93†	$SERO^+$ (EBP)	—	115×115×157	~53000	400	19, 20, 21
6A93†	$PSIL^+$ (EBP)	—	115×115×157	~53000	400	25, 26, 27
6WHA*	—	Gqα‡	115×115×210	~74000	400	34, 35, 36
6WHA*	$SERO^+$ (OBP)	Gqα‡	115×115×210	~74000	400	40, 41, 42
6WHA*	$PSIL^+$ (OBP)	Gqα‡	115×115×210	~74000	400	46, 47, 48
6A93†	—	Gqα‡	115×115×210	~74000	400	31, 32, 33
6A93†	$SERO^+$ (OBP)	Gqα‡	115×115×210	~74000	400	37, 38, 39
6A93†	$PSIL^+$ (OBP)	Gqα‡	115×115×210	~74000	400	43, 44, 45
PMF calculations						
6WHA**	—	Gqα‡‡	115×115×152	~50000	400	—
6A93††	—	Gqα‡‡	115×115×152	~50000	400	—
6WHA**	—	Gqα‡‡‡	115×115×152	~50000	400	—
6WHA**	$SERO^+$ (OBP)	—	115×115×157	~53000	400	—
6WHA**	$PSIL^+$ (OBP)	—	115×115×157	~53000	400	—
6WHA**	$SERO^+$ (EBP)	—	115×115×157	~53000	400	—
6WHA**	$PSIL^+$ (EBP)	—	115×115×157	~53000	400	—
6A93††	$SERO^+$ (OBP)	—	115×115×157	~53000	400	—

$5HT_{2A}R$ model	ligand	transducer	box size/Å^3	no. water	no. lipids	traj. no.
6A93††	$PSIL^+$ (OBP)	—	115×115×157	~53000	400	—
6A93††	$SERO^+$ (EBP)	—	115×115×157	~53000	400	—
6A93††	$PSIL^+$ (EBP)	—	115×115×157	~53000	400	—

* ‘open’/Gqα-bound state. ** ‘open’/Gqα-bound state with position restraints on $5HT_{2A}R$ backbone atoms. † ‘closed’ state. †† ‘closed’ state with position restraints on $5HT_{2A}R$ backbone atoms. ‡ AlphaFold prediction (res 335-359 were replaced with PDB: 6WHA and GDP was placed according to PDB: 7W40). ‡‡ res 335-359 from PDB: 6WHA with distance restraints on Cα atoms. ‡‡‡ AlphaFold prediction without res 335-359 (GDP was placed according to PDB: 7W40) with distance restraints on Cα atoms.
